# Is semirigid ureteroscopy sufficient in the treatment of proximal ureteral stones? When is combined therapy with flexible ureteroscopy needed?

**DOI:** 10.1186/s40064-016-1677-8

**Published:** 2016-01-13

**Authors:** Sadi Turkan, Ozan Ekmekcioglu, Lokman Irkilata, Mustafa Aydin

**Affiliations:** Private Anadolu Hospital, Kastamonu, Turkey; Samsun Training and Research Hospital, Samsun, Turkey; Ozel Anadolu Hastanesi, Beycelebi Mah. Ataturk Cad. No:36/1, Kastamonu, Turkey

**Keywords:** Proximal ureter, Stone, Ureteroscopy

## Abstract

The goals of this study were to examine cases of proximal ureteral stones in which semirigid or flexible ureteroscopes alone were insufficient for endoscopic treatment, requiring the combination of both. A total of 137 patients were retrospectively evaluated. Holmium laser was used as the energy source for stone fragmentation. Each operation was begun with a 6/7.5 Fr semirigid ureteroscope (URS), and continued with a 7.5 Fr flexible URS in those procedures that failed to reach the stone or push-up. Double J stents were inserted into those patients in whom the flexible URS failed. Shock wave lithotripsy (SWL) or a repeat ureteroscopy (after 2–4 weeks) was planned in those patients who were considered to be treated unsuccessfully. The demographic features of the patients, stone sizes, treatment outcomes, need for additional treatment, complications, and the results of the postoperative 1-month early follow-up were evaluated. The mean age of the patients (77 males and 60 females) was 38 ± 6.7 years old, the mean stone size was 12.3 ± 3.7 mm, and the number of patients with persistent hydronephrosis was 86 (62.8 %). A stone-free diagnosis was achieved in a total of 124 patients (90.5 %), using a semirigid URS in 80 patients and a flexible URS in 44 patients. Treatment using a flexible URS was administered in 38 patients (27.7 %) due to push-up, and in 6 patients (4.3 %) because of the failure to advance the semirigid URS into the ureter. The treatment failed in 13 patients (9.4 %) despite the use of both methods. Treatment using low-caliber semirigid ureteroscopy and a holmium laser is possible, regardless of the stone size, in female patients without hydronephrosis. However, the need for combined treatment with flexible ureteroscopy is increased in male patients with hydronephrosis.

## Background

There is no one single treatment method for proximal ureteric stones, but there are several different approaches. Today, extracorporeal shockwave lithotripsy (SWL), ureteroscopy, percutaneous nephrolithotomy (PCNL), antegrade ureterolithotripsy, laparoscopy, and rarely, open surgical procedures are implemented (Wolf 2007). However, SWL and ureteroscopy are considered to be the primary methods for the initial treatment of proximal ureteral stones (Lee 2010). The anatomical structures of the patients, size, localization, and density of the stones, experience of the surgeon, and the availability of the clinical equipment may affect the selection of the treatment method. There have been recent advancements in urological endoscopic devices and imaging techniques, with increases in the options of the energy sources, leading to an increased tendency toward ureteroscopy (Busby and Low 2004). With small-caliber semirigid and flexible ureterorenoscopes and holmium lasers becoming more widespread, outcomes with lower complications and higher success rates are being achieved (Teichman et al. 1997; Scarpa et al. 1999).

Based on the current literature, flexible ureteroscopic applications have been increasing, although the semirigid ureteroscope (URS) is also known to be safe and effective (Karlsen et al. 2007). However, some cases have been encountered in which both tools fail. It is possible to use both tools in combination, with their complementarity increasing their success rates. In this study, we examined cases in which both of these ureteroscopic tools failed in the endoscopic treatment of proximal ureteral stones, requiring combined use. Additionally, we evaluated the extent to which this combined approach can be complementary, and how this affects the results with regard to successful treatment.

## Methods

The files of 137 patients undergoing endoscopic ureteral stone treatment for proximal ureteral stones, between 2010 and 2015, were retrospectively evaluated. The section of the ureter extending from the ureteropelvic junction to the sacroiliac joint was accepted as the proximal ureter. These patients undergoing surgical treatment choose URS treatment primarily among SWL treatment and non of the patients have DJ stent preoperatively. Moreover, the value obtained by the measurement of the long axis of the stone viewed on direct urinary system graphy, ultrasonography, or computed tomography (in millimeters) was accepted as the stone size. Routine biochemistry, urinalysis, and a urine culture antibiogram were performed in all of the patients, and in those patients in which active infections were detected, the procedure was planned after antibiotherapy, when no infection was observed. Prophylactic broad spectrum antibiotic therapy was initiated and continued until postoperative day 5 in all of the patients.

After the administration of general anesthesia, ureteroscopy was begun with a 6/7.5 Fr semirigid URS (Wolf, Germany). Either the Roadrunner^®^ (Cook Medical, Bloomington, IN, USA) or Zebra^®^ (Boston Scientific Corp., Natick, MA, USA) catheter was used as a 0.035–0.038 hydrophilic guidewire for accessing the ureter. The hydrophilic guidewire was the preferred safety wire for use during this procedure.

A Dornier^**®**^ holmium: yttrium-aluminum-garnet laser lithotripter (Ho:YAG) (Germany) was used for the stone fragmentation. In those cases that failed to achieve advancement in the ureter with the semirigid ureteroscope, a flexible ureteroscope (7.5 Fr Karl Storz Flex X2, Tuttlingen, Germany)via an access sheat (12 Fr. Flexor^®^Ureteral Access Sheath,Cook Medical, Bloomington, IN, USA) was used to reach the stone, or pass the stone into the kidney. Neither a device to prevent the migration of the stones nor a balloon dilator used because of the absence in our clinic. The laser lithotripter was used 15 Hz, 0.8 Joule for ureteral stone to dusting and 10 Hz, 1.2 Joule to renal stones for fragmentation if the stone was reached; however, a double J ureteral stent (DJ stent) was inserted and the process was terminated in those cases where the flexible ureteroscope failed to reach the stone. An SWL or repeat URS (after 2–4 weeks) was planned upon the choice of the patient, in those procedures that were considered to be unsuccessful. The creation of stone fragments under 2 mm was considered to be a success, while failure to reach the stone was considered to be unsuccessful. After the procedure, a DJ stent was inserted in some of those patients that underwent semirigid ureteroscopy alone, and all of those patients in whom flexible ureteroscopy was used. After 1 month, the DJ stent was removed under local anesthesia and residual stones size controlled by using direct urinary system graphy and/or urinary system ultrasonography.

The demographic features of the patients, stone sizes, treatment outcomes, need for additional treatment, complications, and the results of the postoperative 1-month early follow-up were evaluated. The statistical analyses were conducted using the Statistical Package for the Social Sciences (SPSS; Chicago, USA), while the categorical variables were compared using the Chi squared test. The continuous variables were compared using the t test. Although there is no need of ethical approval from Ministry of Health for retrospective studies in our country, we get the permission to access patients data by the hospital’s ethical committee.

## Results

The results of the patients were evaluated, and the demographic and stone features of the patients are shown in Table 1.

**Table 1:** Demographics of the patients

Patient characteristics	
Number of patients	137
Gender (male/female)	77/60
Mean age (years)	38 ± 6.7 (21–72)
Preoperative urinary tract infection	22 (16 %)
Stone side	
Right (%)	60 (43.8)
Left (%)	77 (56.2)
Stone size (mm)	12.3 ± 3.7 (7–19)
Presence of hydronephrosis (%)	
Yes	86 (62.8)
No	51 (37.2)

The outcomes of the treatments of the proximal ureteral stones using a semirigid URS only or the combined method can be seen in Table 2. A stone-free diagnosis was achieved in a total of 124 (90.5 %) patients using the semirigid URS in 80 patients, and flexible ureteroscopy in 44 patients. Treatment with flexible ureteroscopy was administered in 38 (27.7 %) patients due to push-up, and in 6 (4.3 %) patients because of tortuosity and stenozis using the semirigid URS. The demographic distribution of the patients undergoing flexible ureteroscopy is shown in Table 3. Maleness, a persistence of hydronephrosis, and push-up significantly increased the need for the use of the flexible URS. In the examination of the stone size, it was found that stones greater than or less than 1 cm did not significantly affect the need to use the flexible URS.

**Table 2:** Outcomes of ureteroscopy for the treatment of upper ureteral stones

	Semirigid alone	Semirigid + flexible	Failures
n (%)	80 (58.4 %)	44 (32.1 %)	13^a^ (9.5 %)
Operation time in minutes	45 ± 9	75 ± 11	42 ± 10
DJ stent insertion n (%)	38/80 (47.5 %)	44 (100 %)	13 (100 %)
Success n (%)	80/137 (58.4 %)	44/57 (77.2 %)	–
Total failure n (%)	–		13 (9.5)
Total success n (%)	124/137 (90.5 %)		–

^a^Image distortion was seen in 2 patients due to hemorrhage. Failed advancement occurred in the ureter in 11 patients

**Table 3:** Demographic distribution of the patients undergoing semirigid + flexible ureteroscopy (combined therapy)

Semirigid + flexible n = 44		p
Reason for flexible ureteroscopy		
Passing of the stone into the kidney	38	0.0210
Failure to reach the stone in the ureter	6	
Gender		
Male	36	0.0239
Female	8	
Hydronephrosis		
Yes	40	0.0172
No	4	
Stone size (mm)		
<1 cm	20	0.681
>1 cm	24	

Of the 13 (9.5 %) patients in whom the treatment failed a DJ stent was inserted, and the process was terminated in 2 patients because of the image distortion due to hemorrhage, and in 11 patients because of the failure to achieve advancement with either ureteroscope, due to tortuosity and stenozis in the ureter. With the exception of hematuria developing in 2 patients in the perioperative period, microperforation in the ureter occurred in 4 patients during the insertion of the stent, and mucosal laceration occurred in 6 patients related to the laser energy. A DJ stent was inserted in these patients, with no problems developing during the postoperative period. Five patients developed high fevers, which lasted one or 2 days in the postoperative period. All of these complications were classified as grade 1 in the Clavien-Dindo Classification of Surgical Complications.

## Discussion

Although SWL has been recommended as the first choice for the treatment of proximal ureteral stones >10 mm, it has recently been replaced with URS, which has been proven to be more effective, creating lower mortality conditions (Cui et al. 2015). The European Association of Urology (EAU) guidelines recommend that the diameter of a semirigid ureteroscope should be less than 8 Fr, reporting that this size can be used in all parts of the ureter (Preminger et al. 2007).

In a study by Dagnone et al. (2005) access to proximal ureteral stones (average size of 1 cm) with a 7.5 Fr semirigid URS was reported to be easy, with a low rate of complications. In a similar study by Kumar et al. (2015) a stone-free diagnosis rate of 86.6 % was reported in the treatment of <2 cm proximal ureteral stones, using a 6/7.5 Fr semirigid URS and holmium laser. The diameter of the semirigid URS which was used in this study was 6/7.5 Fr, with no significant challenges being encountered in accessing the stones in the majority of the patients. The operation was converted to a flexible URS in 6 patients in whom the semirigid URS could not be advanced, due to reasons such as tortuosity and stenozis.

In a study by Wu et al.(2005) comparing the SWL and 6/7.5 Fr semirigid URS for the treatment of proximal ureteral stones in 220 patients, in terms of safety and cost-effectiveness, the semirigid ureteroscopic procedure was proposed to be the first line of treatment . However, it is known that the cost of flexible URS equipment is much higher when compared to the semirigid URS, and this tool can easily malfunction. The cost of the flexible URS has been reported to be $1116.00 (USD) for each patient, with the use of additional equipment (Gurbuz et al. 2014), while the advantages of the semirigid URS include its wider working channel and better images obtained using high irrigation. However, the flexible URS can be difficult to manipulate and insert into the ureteral orifice, with its smaller optic diameter and working channel, and its longer operation time (Miernik et al. 2014). Therefore, the use of a flexible URS in terms of accuracy could be more efficient as well as contribute to a prolonged economic life and decreased treatment costs.

Longer urethras and fixed prostatic urethras in men may make maneuvering and access to proximal ureteral stones in semirigid ureterorenoscopy difficult. The easier mobilization of the urethra and the ureter, with a lower degree of curvature in the transverse iliac in women, facilitates the URS procedure. In our study, 36 of 44 patients in whom treatment with the semirigid URS failed, and the flexible URS was applied, were consistent with this anatomical situation.

Retropulsion of the stone into the renal pelvis may occur during ureteroscopy because of the dilatation proximal to the stone, strong retrograde fluid pressure, and impulsion power which the energy source exerts on the stone. However, in those cases of push up, it is possible to continue the process in an uninterrupted fashion with a flexible URS. In a study by Gupta et al. (2007) the rate of retropulsion due to the use of a holmium laser was reported to be 3.3 % in 208 patients, 55 of which had proximal ureteral stones. In our study, the procedure was converted to flexible ureteroscopy in 38 (27.7 %) patients due to push up. We believe that dilatation has a marked effect on this rate, and was related with the small number of proximal ureteral stones in the previously mentioned study. It is expected that the conversion to a flexible URS is more likely in the endoscopic treatment of proximal ureteral stones with prominent hydronephrosis.

In our study, a Ho:YAG laser was used as the energy source in all of the patients, and is considered to be safe and effective in intracorporeal lithotripsy, with rather low complication rates (Jiang et al. 2008). In this study, no major complications were seen due to the use of the laser. It has been previously suggested that a semirigid URS with pneumatic lithotripsy may be a good alternative in the absence of a flexible URS with a holmium laser. For example, Khairy-Salem et al. (2011) reported a success rate of 98 % using pneumatic lithotripsy and a semirigid URS in proximal ureteral stones, when the flexible URS was not available. However, considering what may occur during endoscopic stone surgery, it is ideal to have several types of equipment available to complete the procedure in a single session. Therefore, it would be more appropriate to consider the flexible URS to be complementary, rather than an alternative to the semirigid URS. In addition, Khoder et al. (2014) reported a success rate of 75.4 % using a combined approach, composed of a 7/9.5 Fr semirigid URS and flexible URS in the endoscopic treatment of proximal ureteral stones. In our study, this rate was determined to be 32.1 %, possibly because of the use of a small-caliber semirigid URS. Moreover, Süer et al. (2015) stated that they treated 48 of 88 patients with renal pelvis stones using a 9.5 Fr semirigid URS, and 40 patients in whom the stones could not be accessed (or they could not produce adequate fragmentation) with a flexible URS, achieving a stone-free rate of 85 %.

In our study, the total stone-free diagnosis rate, using the combined procedure in a single session, was found to be 90.5 %. In a previous study comparing the combined endoscopic approach with the semirigid URS alone, the combined approach was reported to be more cost-effective by decreasing the need for an additional procedure (Goldberg et al. 2013). Additionally, there are publications reporting that the treatment of proximal ureteral stones with a semirigid URS has a higher complication rate and lower success rate when compared to the flexible URS (Yencilek et al. 2010; Perez Castro et al. 2014). None of our patients developed major perioperative or post-operative complications. Of the twelve patients that developed hematuria, microperforation, or mucosal lacerations in the perioperative period were treated with the insertion of DJ stents.

Some limitations of our study included not considering the body mass indices of the patients, not specifying the degree of hydronephrosis in the patients who developed push up, not taking into account the experience of the surgeon and the cost effectiveness, and not examining the comorbidity factors of the patients in whom the treatment failed. Further studies with larger series evaluating the level of the stone in the ureter, degree of the dilatation being caused, and the experience of surgeon, using multivariate analyses, are needed.

## Conclusion

Regardless of stone size, treatment using low-calibration semirigid ureteroscopy with a holmium laser is possible in female patients without hydronephrosis. In addition, low complication rates and high success rates can be achieved in male patients, especially those with hydronephrosis, using combined therapy with a flexible ureteroscope. Therefore, flexible URS should be considered as complementary to, rather than an alternative for, semirigid URS in the endoscopic treatment of proximal ureteral stones.
